# Multiple Species Comparison of Cardiac Troponin T and Dystrophin: Unravelling the DNA behind Dilated Cardiomyopathy

**DOI:** 10.3390/jcdd4030008

**Published:** 2017-07-07

**Authors:** Jennifer England, Siobhan Loughna, Catrin Sian Rutland

**Affiliations:** 1 School of Life Sciences, Medical School, Queens Medical Centre, University of Nottingham, Nottingham NG7 2UH, UK; jennifer.england88@gmail.com (J.E.); Siobhan.loughna@nottingham.ac.uk (S.L.); 2 School of Veterinary Medicine and Science, University of Nottingham, Sutton Bonington Campus, Sutton Bonington, Leicestershire LE12 5RD, UK

**Keywords:** dilated cardiomyopathy, troponin, dystrophin, mutation, species

## Abstract

Animals have frequently been used as models for human disorders and mutations. Following advances in genetic testing and treatment options, and the decreasing cost of these technologies in the clinic, mutations in both companion and commercial animals are now being investigated. A recent review highlighted the genes associated with both human and non-human dilated cardiomyopathy. Cardiac troponin T and dystrophin were observed to be associated with both human and turkey (troponin T) and canine (dystrophin) dilated cardiomyopathies. This review gives an overview of the work carried out in cardiac troponin T and dystrophin to date in both human and animal dilated cardiomyopathy.

## 1. Introduction to Cardiomyopathies

Cardiomyopathies are a group of diseases of the heart muscle that contribute to cardiac dysfunction leading to heart failure [1]. They are associated with a high rate of morbidity and mortality and increased risk of sudden cardiac death. According to the American Heart Association, there are five classifications of cardiomyopathy: dilated cardiomyopathy (DCM), hypertrophic cardiomyopathy (HCM), restrictive cardiomyopathy (RCM), arrhythmogenic right ventricular cardiomyopathy (ARVC), and left ventricular non-compaction (LVNC) [1,2]. HCM in humans is characterized by left and/or right ventricular wall thickening with non-dilation of the ventricles with myocyte disorganization, fibrosis, increased calcium sensitivity, and cardiac arrhythmias [3]. RCM, a rare form of cardiomyopathy, presents with dilated atria, restricted ventricular filling, reduced diastolic volume in the ventricles with occasional fibrosis of the myocardium [4]. Arrhythmogenic right ventricular cardiomyopathy is associated with cardiomyocyte replacement with fibrofatty tissue. This fatty deposition results in obstruction of electrical conduction, thus resulting in arrhythmias [5]. LVNC is characterized by the presence of prominent trabeculae and deep recesses in the ventricular cavity of the mature heart and can coexist with congenital heart defects such as atrial and ventricular septal defects, aortic stenosis and aortic coarctation [6]. These cardiomyopathies are also further subclassified into inherited and acquired diseases. Mutations in sarcomeric proteins have been associated with all of the cardiomyopathies, except for ARVC.

### 1.1. Dilated Cardiomyopathy in Humans

Idiopathic dilated cardiomyopathy has a high incidence of 1 in 250 people and is the most common reason for heart transplants [2]. The DCM phenotype includes dilation of one or both ventricles and systolic dysfunction, and is usually associated with congestive heart failure [7,8]. DCM is associated with a range of causes such as familial, environmental, idiopathic, or as part of the progression of other cardiovascular disease [9]. Genetic forms of DCM, which account for 30–50% of cases, usually result from mutations in genes encoding cytoskeletal and sarcomeric proteins such as myosin heavy chain 7 (*MYH7*), cardiac muscle troponin T (*TNNT2*), and Titin (*TTN*) [10].

### 1.2. Dilated Cardiomyopathy in Animals

Clinical data indicates that DCM accounts for 10% of cardiac diagnoses in dogs, with cardiovascular disease being the fourth most common cause of canine death [11,12]. In dogs, differing incidence rates in the breeds have indicated that a genetic link may be present. An autosomal link was first suggested in the 1990s [13]. Since then a number of genetic associations have been made throughout the breeds [14]. These have ranged from single gene deletions [15] to splice site and point mutations [16,17,18]. More recently models and genetic testing in affected dogs has indicated the role of both multiple allele linkage and multiple allele involvement in DCM [19,20].

DCM has been reported in wild turkeys [21], furazolidone-induced DCM captive turkeys [22,23,24], hypoxia-induced hypoxemia birds [25] and calcium activated turkey models [26]. Roughly 2–5% of domesticated turkeys get DCM within the first four weeks of hatching [27]. Diet has been shown to play a key role with birds fed a high protein diet twice as likely to suffer death as a result of cardiomyopathy [28], but variations in Troponin T and phospholamban (PLN) have also been linked to DCM [27,29].

Feline DCM clinical outcomes improved rapidly following the discovery that dietary taurine supplementation worked in both naturally acquired and experimental populations [30,31,32,33]. Despite this discovery, DCM was still present in the population and investigations into two breeding colonies indicated that a genetic factor was likely [34]. In addition to these species, cattle and chickens also have DCM [35,36,37].

A recent review highlighted that both cardiac troponin T and dystrophin mutations play a role in cardiomyopathies in both humans and animals [38]. This review explores the literature to date and the mutation links to DCM for each gene.

### 1.3. Expression, Structure, and Function of Cardiac Troponin T

The troponin complex is a multipart protein found in the thin filament of the sarcomere. It is composed of three parts: troponins (Tn) C, I, and T encoded by *TNNC*, *TNNI*, and *TNNT*, respectively [39]. TnC, binds Ca^2+^ which induces a conformational change to the Tn complex [40]. TnI is the inhibitory component of the unit. Muscle contraction is powered by the actin-myosin motor, which converts ATP into energy (using ATPase) for a power stroke through each cross-bridge cycle [40]. TnI blocks ATPase activity in a Ca^2+^ dependent manner.

TnT is essential for structural integrity of the troponin complex, binding tropomyosin (TPM), TnC, and TnI and is essential for sarcomere assembly and cardiac contractility [41]. When Ca^2+^ binds to TnC inducing a conformational change to the Tn complex, cTnC binds to cTnI causing it to release cTnT. This lever action moves TPM around the actin filament, thus exposing myosin binding sites on the actin filament.

Three TnT genes exist, slow skeletal TnT (*TNNT1*), cardiac TnT (*TNNT2*), and fast skeletal TnT (*TNNT3*). In addition, alternative RNA splicing adds another dimension of generating multiple isoforms of TnT, a process with is developmentally regulated. Exon 4, exon 5, and exon 13 are alternately spliced, where exon 5 is absent in the adult isoform of cTnT. This exon produces a highly acidic and negatively charged 10 amino acid segment which shows higher Ca^2+^ sensitivity of the ATPase activity and force production when compared to the adult isoform [42], suggesting not just a structural role, but also a functional one for TnT in the sarcomere.

**Figure 1:**
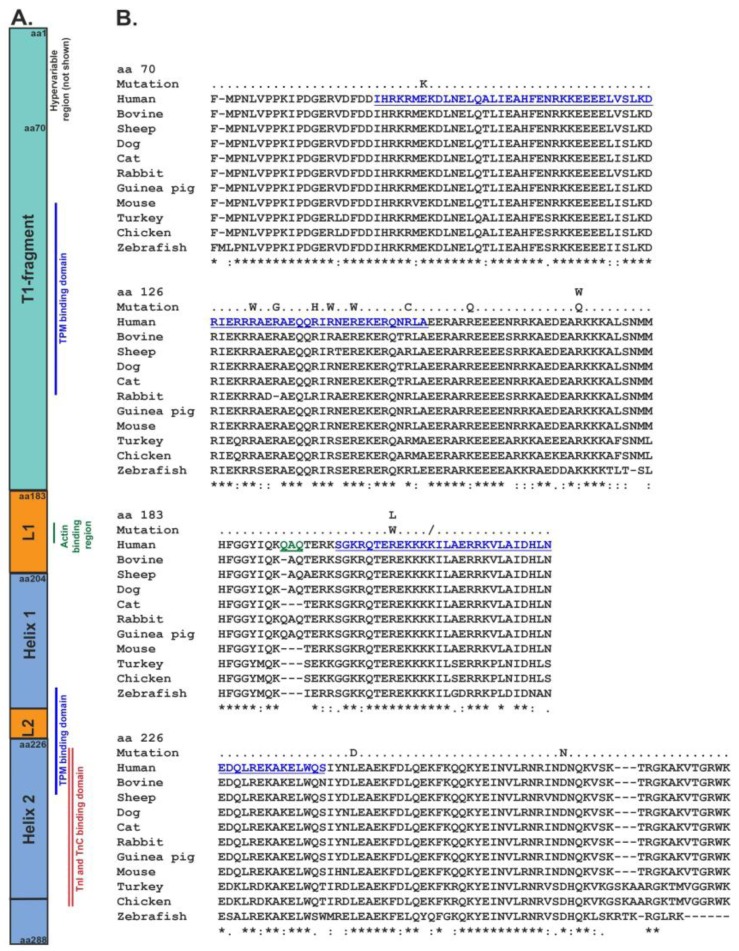
The structure and functional regions of cardiac troponin T alongside the sequence alignment of the regions. (**A**) A schematic image showing the structure of the cTnT protein. The first region of the T1-fragment is known as the hypervariable region and is highly unconserved. Different binding domains of other thin filament proteins are highlighted in the schematic, the blue line indicates the tropomyosin binding domain, green the actin binding domain, and red is the troponin I and troponin C binding domains. L1 and L2 indicate the flexible linkers between helix zone 1 and 2; (**B**) A multiple species alignment of the cTnT protein in comparison to the regions shown in A. Mutations associated with dilated cardiomyopathy are aligned also. The first 70 amino acids of cTnT have been excluded as no mutations are known in this area. Accession numbers for each species are: human NP_001001430, bovine AF175558, sheep P50751, dog NP_001003012, cat NP_001009347, rabbit A25345, guinea pig NM_001172863, mouse NM_011619, turkey AF274301, chicken NM10013, and zebrafish NP_69085.

Understanding the structure of cTnT may elucidate a reason why point mutations cause such devastating effects on the heart. At the N-terminus of cTnT, the first 1–59 amino acid residues are enriched with negatively charged residues of glutamine and aspartate [43]. It is a highly variable region, lacking any known protein binding sites, such as actin or tropomyosin [44]. Conversely, the remaining N-terminus, middle portion and C-terminus of the protein are highly conserved and enriched with positively charged residues (Figure 1A,B) [43]. There are two TPM binding domains, one of which is present at the N-terminus (known as T1 fragment) within residues 98–138 [45]. The T1 sub-fragment is thought to bind to the C-terminus of the TPM in the region where TPM overlaps head to tail to form a continuous TPM filament [46]. Residues 183–204 act as a flexible linker between the T1-fragment and the C-terminus [47]. An actin binding site is present in this linker. The C-terminus of TnT is composed of α-helical rings at residues 204–220 (Helix 1) and 226–272 (Helix 2) [47,48]. A second TPM binding domain (T2) is close to the C-terminus, but controversy remains as to its precise location (either at residues 197–239 or the last 16 residues of the TnT sequence) [45,49,50,51,52].

### 1.4. Cardiac Troponin T Mutations Relating to Cardiomyopathy in Humans

Currently, over 90 mutations have been identified in Tn subunits associated with hypertrophic cardiomyopathy, dilated cardiomyopathy, left ventricular non-compaction, and restrictive cardiomyopathy [53,54], with mutations in cTnT believed to have a frequency of 3–6% in DCM [55]. Mutations in cTnT that have been linked with DCM are listed in Table 1 along with their clinical presentation and known molecular and cellular effects. These mutations are also compared to multiple species for conservation in Figure 1B. Point mutations are most commonly found in the conserved region of the T1 terminal and the C-terminal of cTnT. Interestingly, no mutations have been found in the N-terminal hypervariable region of cTnT. This may be due to the nature of such a variable region in that it is more tolerable to changes introduced by a single amino acid substitution than an area that is highly conserved would be.

Mutations associated with HCM and RCM generally lead to increased Ca^2+^ sensitivity in the thin filament [53]. In contrast, decreased Ca^2+^ sensitivity is commonly observed in mutations in cTnT associated with DCM (R131W, R139H, R141W, R151C, R159Q, R205W, ΔK210, and K273E) [52,56]. Lu et al. suggested that the reason one mutation could possibly have such a huge impact on Ca^2+^ sensitivity was due to an increase in the affinity of cTnT for TPM1 observed for the R141W mutation. The mutation strengthened the integrity of cTnI in the thin filament by stabilizing the interaction between cTnT and TPM, which might allow cTnI to inhibit the thin filament more effectively, leading to Ca^2+^ desensitization [57]. However, some mutations report no change (R134G) [52], suggesting that Ca^2+^ sensitivity is a stimulus sufficient to cause DCM, but not essential, or the only cellular mechanism triggering cardiac remodeling observed in DCM.

Mutations within the same contractile protein can cause HCM, RCM, and DCM, with each cardiomyopathy having its own distinct phenotype. This suggests that different signaling pathways or a graded response within the same pathway is activated, thus producing such variable phenotypes. For example, in transgenic mice with a truncated myosin binding protein-C (MyBP-C) protein, there is a graded response with the heterozygous mouse developing HCM and the homozygous developing DCM [77]. In addition, a heterozygous mutation in TnI results in RCM, while the homozygous state results in DCM [78,79]. Concurrently, the ratio of mutated to wildtype transcripts is critical in determining severity of DCM [80,81]. On the other hand, molecular signaling studies in DCM and HCM mouse models have reported activation of different signaling pathways suggesting remodeling and profibrotic mechanisms in the two cardiomyopathies are differentially regulated, thus producing two separate phenotypes [82].

Left ventricular systolic function is compromised in DCM, a key symptom for the disease; however, this is usually preserved in HCM. Interestingly, in the latter stages of HCM morphological features resembling DCM occurs in 5–10% of patients. Patients have a progression to systolic impairment associated with left ventricular remodeling, wall thinning and cavity dilation, thus resembling DCM. Therefore, it is known as the dilated phase of HCM (or D-HCM) [83]. D-HCM is more symptomatic than DCM, where the left atrium is also larger in size with a higher prevalence of atrial fibrillation. This is also combined systolic and diastolic dysfunction. In addition, left ventricular remodeling is not seen in D-HCM and the prognoses is much poorer [83]. Mutations in *TNNT2* have been associated with this HCM to D-HCM transition, I79N, R92W, R92Q, R113W, and K273E (Table 2) [71,84,85,86,87,88,89,90].

### 1.5. Cardiac Troponin T Mutations Relating to Cardiomyopathy in Non-Humans

As mentioned above, alternative splicing of *TNNT2* gives rise to various isoforms that are developmentally regulated. Each isoform varies in structure and function, thus fine-tuning muscle contractility. Alternative spliced isoforms, independent of developmental regulation, have been found in diseased hearts and additionally have been found to cause DCM in turkeys and dogs [92]. A low molecular weight cTnT, due to the exclusion of exon 8, is expressed in turkeys (often induced by Furazolidone). Exon 8 accounts for 12 amino acids in the protein and expression of this low molecular weight isoform over 30% in myofibrils results in changes to the conformation and the binding affinity for TnI and TPM, with minor alterations to Ca^2+^ sensitivity [23].

Exon 7 deletion is observed in canine DCM, where exon 7 is the mammalian equivalent of avian exon 8. The 12 amino acid deletion in canine (Doberman pincher) showed reduced shortening and re-lengthening of muscle fibers upon stimulation [92]. Exclusion of Exon 6 in guinea pig results in a 25 amino acid residue difference, much larger than that seen in turkey and canine and would suggest significant functional effects [92].

### 1.6. Expression, Structure, and Function of Dystrophin

The dystrophin (*DMD*) gene codes for the dystrophin protein and at 2.3 megabases long, with 86 exons, it is also one of the largest in the human genome and is situated on the X chromosome [93,94,95]. The isoforms expressed in cardiac tissue are brain (Dp427 B), muscle (Dp427 M), retinal (Dp260, low expression levels), and G-dystrophin (Dp71) [96,97]. However, the muscle promoter is the main one used for cardiomyocyte expression, with further isoforms produced via splicing alterations such as exon scrambling and exon skipping [98,99].

### 1.7. Dystrophin Mutations Relating to Cardiomyopathy in Humans

Dystrophin has been of interest for many years as mutations have been linked with Duchenne and Becker muscular dystrophies, X-linked DCM and tumor progression and development [99,100,101,102]. A basic PubMed search (June 2017) shows 2526 articles with the word ‘dystrophin’ in the title alone, and the same search in ‘Web of Science’ shows 3484 results. The Leiden Open Variation Database shows 25,828 confirmed DNA variants and 25,830 RNA and protein variants for human DMD and by 2005 there were 4704 known mutations [103,104]. The UMD-DMD France mutations database reports 2898 fully characterized mutations in dystrophin causing either Becker muscular dystrophy, Duchenne muscular dystrophy, or X-linked DCM. It is estimated that around 33% of all Becker muscular dystrophy and Duchenne muscular dystrophy causing mutations are spontaneous [105]. Several reports have shown no linkage between the size of deletion or duplication and clinical severity. Clinical outcome does appear to be correlated to whether frameshift or nonsense-mediated RNA decay occurs, indeed deletions of up to 50% of dystrophin have resulted in Becker muscular dystrophy [106,107,108,109,110]. The mutation types and clinical outcomes were reviewed in detail by Muntoni and colleagues [97].

One of the biggest problems is determining which mutations cause cardiac only symptoms, as even most of the X-linked cardiomyopathy mutations result in some degree of skeletal muscle problems, such as increased plasma creatine levels. In contrast, many of the human and animal cardiomyopathies occur without concomitant skeletal muscle disorders. However, a detailed analysis of the X-linked cardiomyopathy mutations in humans is explored by Ferlini and colleagues [111], but more than 30 mutations in Duchenne muscular dystrophy are thought to cause DCM. There are some common areas which are affected though. There are a group of mutations in the 5′ region that result in altered transcription and splicing. It is thought that the skeletal muscle is able to compensate for this isoform by upregulating other isoforms, but this is not possible in the cardiac tissue [99,112]. Loss of transcript has been shown in a number of cases where a mutation has affected exon 1 resulting in loss of the M isoform [99,113,114]. Not all mutations result in full loss of isoforms. One 5′ mutation (duplication of exons 2–7) showed normal transcription in skeletal and cardiac muscle, but a lack of protein expression in the heart tissue only [115]. An intron 11 deletion resulted in a lack of the all transcripts in cardiac tissue, whereas skeletal muscle was only partially affected, with normally spliced isoforms able to be expressed [111]. Interestingly, other splice mutations in the 5′ end result in dystrophin expression in both cardiac and skeletal muscle [116]. A mutation in exon 9 also indicated a cardiac specific role for the epitope in question as skeletal effects were not observed [117]. These cardiac affecting mutations highlight the importance of the differing tissue types when considering both transcript and protein levels and their affects in differing regions.

The mutation region also appears to correlate relatively well with clinical severity. In patients with 5′ mutations, the clinical manifestations are usually severe, whereas those in the spectrin-like domain present with a less severe phenotype. It has been hypothesized that the 5′ region mutations may affect enhancers, whereas the spectrin-like domain mutations may only cause loss of a cardiac relevant domain, such as the hinge domain or a regulating sequence. The full mechanisms have yet to be elucidated [111,118].

### 1.8. Dystrophin Mutations Relating to Cardiomyopathy in Non-Humans

Another problem when looking at the murine models is the differing phenotypes between humans and mouse models. In general, the lines created which affected dystrophin only showed very mild phenotypes and usually later on in life [119,120,121]. The symptoms become more pronounced when using double knockdown models including integrin/dystrophin, utrophin/dystrophin, myoD/dystrophin, and δ-sarcoglycan/dystrophin [122,123,124]. In addition, these mice frequently are easier to work on, as the cardiomyopathy symptoms occur earlier in life in comparison to dystrophin only models, making the work less expensive and less time consuming [125]. Over 60 models of Duchenne muscular dystrophy have been published and consist of both naturally occurring and laboratory induced animals ranging from the fly, cat, pig, mouse, and dog [126,127]. Despite dystrophin being the primary target gene in many (but not all) of these models and cardiomyopathy being the most common cause of death, very few have DCM as the only symptom, despite so many animals naturally occurring with DCM only in normal populations.

The dog has been a model for Duchenne muscular dystrophy since 1951 [128]. Later groups started investigating the dystrophin gene with over 20 breeds reported as having dystrophin disruption [126]. Research in the 1990s showed that dystrophin was important in canine X-linked muscular dystrophy and dilated cardiomyopathy [129,130,131]. A point mutation in Golden retrievers in intron 6 (consensus splice acceptor) resulted in a muscular dystrophy like phenotype [132,133]. The resulting colonies were used for phenotypic evaluation and genetic therapy trials for a number of years [132,134]. Gene therapy in both mouse models and dogs have shown promising results for delivering mini- or micro-dystrophins delivered via adeno-associated viral vectors especially when immunosuppression is used [135,136,137]. Although this is more suitable for skeletal muscle, cardiovascular tissue is more complex. A trial using intravenous injection of adeno-associated virus serotype-9 did not show cardiac muscle transduction [138], a serious problem when considering DCM treatment. The first work to look at DCM in three breeds (Doberman Pinschers, Irish Terrier, and German Shorthaired Pointers) indicated that the dystrophin promoter region was not involved in DCM for the first two breeds, but a deletion was observed in the German Shorthaired Pointers. It is worth highlighting that the numbers of Doberman Pinschers and Irish Terriers were relatively small (*n* = 9 and 1 respectively) [129]. Dystrophin mutations are also complex in that mutations are frequently spontaneous. It has been indicated that some breeds and families are more likely to develop DCM [12,139,140,141,142,143]. There is a strong suggestion in the literature for an autosomal inheritance mode in canine breeds such as the Irish Wolfhound, Newfoundland, Great Dane, and Portuguese water dog [139,140,141,142,143,144]. These indicate that dystrophin, with its one third *de novo* mutation rate and X-linked DCM, may not play a role in many canine DCM cases, but with around 5% of human DCM cases presenting as X-linked, this mode is still a possibility in other species [145].

## 2. Discussion

Dilated cardiomyopathy has been increasingly of concern in both domestic and commercial animals. The economic loss, food security threats and impact of loss and treatment costs make the condition important to both breeders and owners, the veterinary medicine community, healthcare insurance, pharmaceutical companies, and those involved in the food industry. Advances in genetic testing and more recently gene therapy proof of concept advances are now making mutation detection more accessible and treatment development more promising even in the large dystrophin gene [146,147,148]. In addition, finding causative mutations enables testing for at risk breeds and species prior to clinical detection and/or symptoms. The decreasing costs of mutation detection and genetic testing tools mean that undertaking this approach in non-human animals are more viable options than a few years ago. Given the size and complexity of dystrophin, investigating the mutation rate will be more expensive and time consuming that the smaller cardiac troponin T. In addition, although many murine and canine dystrophin mutations have shown phenotypic alterations, few are DCM only, which makes it difficult in assessing whether the gene is valuable as a DCM only candidate. With the high *de novo* rate of mutation and the emerging evidence that DCM in dogs for example is an autosomal inherited disorder in general, it may reduce the possibility that the gene is involved in some species. However, when considering that there are over 4500 mutations described in humans and only 20 in the dog, and that DCM only causing mutations are rare in humans [149,150], it is possible that mutations in non-human animals could cause DCM alone, or DCM accompanied by minimal skeletal disruption.

## 3. Conclusions

Both dystrophin and cardiac troponin T are likely to show regions of interest in relation to DCM given the similarities and conservation between the human and animal sequences. The two genes have differing functions within the cell, but are linked between the species by both sequence and cellular mechanism similarities and by the fact that mutations in each gene have been shown to cause DCM in both humans and non-humans. Many of the cardiac troponin T mutations that cause DCM in human cases result in altered calcium sensitivity, which is also likely to affect animals as it is a common feature of many DCMs [52,56,151]. Therefore, despite the aforementioned caveats, both genes make good candidates for undertaking mutation detection studies in species affected by DCM. In the long term this may help with diagnosis and treatment of affected veterinary patients and assist with breeding programs in animals.

## Figures and Tables

**Table 1:** Mutations found in *TNNT2* associated with dilated cardiomyopathy, sudden cardiac death (SCD), dilated cardiomyopathy (DCM), hypertrophic cardiomyopathy (HCM), Leiden Muscular Dystrophy (LOVD).

Mutation/rs ID	Exon	Clinical Presentation/Frequency	Molecular/Cellular Effects	Reference
E96K/LOVD#0030558	10	Familial DCM (age 5 months)	-	[58,59]
R131W/rs 483352833	11	DCM diagnosed (age 23); SCD (age 16); heart failure death (age 34)	Enhanced cTnC-cTnI interaction while decreasing cTnC-cTnT interactions; decreased Ca^2+^ sensitivity; decreased ATPase activity	[56,60,61]
R134G/45525839	11	Familial DCM (age 6) and heart transplant (by age 7)	increased maximal force development; no change in Ca^2+^ sensitivity	[52,58,60,62]
R139H	11	Late onset DCM (age 70)	Decreased Ca^2+^ sensitivity and maximal force development	[63]
R141W/rs 7315379 and rs 74315380	11	Idiopathic DCM and Familial DCM; does not cause SCD	Increased affinity of cTnT to TPM; decreased Ca^2+^ sensitivity; decreased ATPase activity	[56,62,64]
R144W/rs 483352832	11	Familial DCM with history of SCD; variability in severity within the family	Decreased ATPase activity	[65]
R151C/rs 45608937	11	Idiopathic DCM	Decreased Ca^2+^ sensitivity	[52,60]
R159Q/rs 45501500	12	Idiopathic DCM	Decreased Ca^2+^ sensitivity	[52,60]
A171S	12	Familial DCM and SCD (>age 20)	-	[66,67]
R173G	12	Familial DCM. Diagnosed at birth with dilated left ventricles (*n* = 2). Asymptomatic maternal uncle (age 45) and his cousin with mildly dilated left ventricle.	-	[68]
R173W	12	Familial DCM with dilated left ventricle, decreased ejection fraction and heart transplant (age 14)	Altered Ca^2+^ regulation; decreased contractility; sarcomere disorganization	[69,70]
R173Q	12	Dilated left ventricle at birth; SCD due to arrhythmia; asymptomatic dilated left ventricle	-	[68]
R205W/rs 45586240	14	Idiopathic DCM (6 months)	Decreased Ca^2+^ sensitivity	[52,60]
R205L/rs 121964860	14	Familial DCM	Impaired cTnI-cTnC and cTnC-cTnT interactions; decreased ATPase activity	[56,61]
ΔK210/rs 121964859	14	Familial DCM; high incidence of SCD	Decreased Ca^2+^ sensitivity, maximal force and ATPase activity; impaired cTnC-cTnI and cTnC-cTnT interactions	[61,71,72,73,74]
E244D/rs 45466197	15	Familial DCM, previously associated with HCM; idiopathic DCM (age 7), heart transplant required; mutation seen with A277V mutation in TPM1. Identified in one family.	-	[52,58,75]
D270N/rs 121964861	16	Familial DCM (early death in *n* = 2 family members age 44 and 21)	Impaired cTnC-cTnI and cTnC-cTnT interactions; decreased Ca^2+^ sensitivity and ATPase activity	[61,76]

**Table 2:** Mutations in *TNNT2* associated with multiple cardiomyopathies, including hypertrophic cardiomyopathy (HCM), dilated cardiomyopathy (DCM), restrictive cardiomyopathy (RCM), sudden cardiac death (SCD).

Mutation	Exon	Clinical Presentation	Molecular/Cellular Effects	Reference
I79N	8	Previously diagnosed in HCM with high incidence of SCD. Idiopathic DCM (age 68 and 64)	Disrupts the TPM binding domain of cTnT	[86,87]
R92W	10	Progression from HCM to DCM	-	[71,84]
R92Q	10	Mixed phenotype such as mild HCM, DCM with ventricular dysfunction and noncompaction; severe left ventricular dysfunction, dyspnea, chest pain and SCD (and SCD without clinical manifestation)	-	[88,89]
R113W	10	HCM, DCM and RCM	-	[62,90,91]
K273E	16	Transition from FHC to DCM during disease progression; initially asymmetrical septal hypertrophy with disease progression to DCM; high incidence of SCD	Decreased ATPase activity and Ca^2+^ sensitivity, impaired force production	[85]
